# An Informative Review of Radiomics Studies on Cancer Imaging: The Main Findings, Challenges and Limitations of the Methodologies

**DOI:** 10.3390/curroncol31010027

**Published:** 2024-01-10

**Authors:** Roberta Fusco, Vincenza Granata, Igino Simonetti, Sergio Venanzio Setola, Maria Assunta Daniela Iasevoli, Filippo Tovecci, Ciro Michele Paolo Lamanna, Francesco Izzo, Biagio Pecori, Antonella Petrillo

**Affiliations:** 1 Medical Oncology Division, Igea SpA, 80013 Naples, Italy; r.fusco@igeamedical.com; 2 Division of Radiology, Istituto Nazionale Tumori IRCCS Fondazione Pascale—IRCCS di Napoli, 80131 Naples, Italys.setola@istitutotumori.na.it (S.V.S.); m.iasevoli@istitutotumori.na.it (M.A.D.I.); f.tovecci@istitutotumori.na.it (F.T.); cmp.lamanna@istitutotumori.na.it (C.M.P.L.); a.petrillo@istitutotumori.na.it (A.P.); 3 Division of Epatobiliary Surgical Oncology, Istituto Nazionale Tumori IRCCS Fondazione Pascale—IRCCS di Napoli, 80131 Naples, Italy; f.izzo@istitutotumori.na.it; 4 Division of Radiation Protection and Innovative Technology, Istituto Nazionale Tumori IRCCS Fondazione Pascale—IRCCS di Napoli, 80131 Naples, Italy; b.pecori@istitutotumori.na.it

**Keywords:** biomedical imaging, radiomics, machine learning, deep learning

## Abstract

The aim of this informative review was to investigate the application of radiomics in cancer imaging and to summarize the results of recent studies to support oncological imaging with particular attention to breast cancer, rectal cancer and primitive and secondary liver cancer. This review also aims to provide the main findings, challenges and limitations of the current methodologies. Clinical studies published in the last four years (2019–2022) were included in this review. Among the 19 studies analyzed, none assessed the differences between scanners and vendor-dependent characteristics, collected images of individuals at additional points in time, performed calibration statistics, represented a prospective study performed and registered in a study database, conducted a cost-effectiveness analysis, reported on the cost-effectiveness of the clinical application, or performed multivariable analysis with also non-radiomics features. Seven studies reached a high radiomic quality score (RQS), and seventeen earned additional points by using validation steps considering two datasets from two distinct institutes and open science and data domains (radiomics features calculated on a set of representative ROIs are open source). The potential of radiomics is increasingly establishing itself, even if there are still several aspects to be evaluated before the passage of radiomics into routine clinical practice. There are several challenges, including the need for standardization across all stages of the workflow and the potential for cross-site validation using real-world heterogeneous datasets. Moreover, multiple centers and prospective radiomics studies with more samples that add inter-scanner differences and vendor-dependent characteristics will be needed in the future, as well as the collecting of images of individuals at additional time points, the reporting of calibration statistics and the performing of prospective studies registered in a study database.

## 1. Introduction

Cancer presents an exclusive medical decision-making environment when considering its multiple forms during the disease course, the patient’s situation, available treatment options and treatment response. Technological developments in oncology imaging offer advantages in addressing the challenges associated with accurately detecting, characterizing and monitoring cancer, but conventional imaging assessment of cancer classically relies on visual assessments whose interpretations can be enhanced by innovative computational techniques. Radiomics promises major progress in the quantitative interpretation of images.

Radiomics is the analysis of medical images to obtain multiple quantitative data that cannot be identified by the human eye [1,2,3,4,5,6,7,8]. It provides insight into underlying pathophysiological phenomena not accessible to simple visual analysis.

Radiomics can be divided into two categories [7]: handcrafted radiomics and deep learning-based radiomics. The conventional radiomics workflow is typically based on extracting predesigned “features” (also referred to as handcrafted or engineered features) by a segmented region of interest (ROI). Nevertheless, recent advancements in deep learning have inspired trends toward deep learning-based radiomics (DLRs), which is also referred to as discovery radiomics.

In medicine, handcrafted radiomic models use data analytics to extract many features from medical images and is made up of several steps: (1) segmentation of the target lesion with manual segmentation by radiologists or with automatic and semi-automatic tools, (2) feature extraction to obtain multiple quantitative metrics and parameters from medical images, (3) feature selection with the aim of reducing the number of extracted features by avoiding correlated or redundant metrics, (4) analysis/classification by creating a predictive model using machine and deep learning approaches and (5) the validation of the results [9,10,11,12,13,14,15,16,17,18].

The extracted radiomics parameters can be morphological or statistical, and can be of first-order, second order and/or higher-order statistics. The morphological features characterize the target’s segmented lesion shape and its geometric features. Statistical features define the individual voxel values distribution, the associations between neighboring voxels allowing for extraction from medical image features linked to lesion heterogeneity and the quantification of successive voxels with equal intensities along certain directions. Higher order statistical metrics are acquired through the application of filters or mathematical transformations to the images [18,19,20,21,22,23,24,25,26,27,28,29,30,31,32,33].

Radiomics could be associated with clinical, pathological or genetic data to provide a model with predictive ability in order to offer a tailored precision medicine using these features as input of pattern recognition and artificial intelligence [34,35,36,37,38,39,40,41,42,43,44,45,46,47,48,49,50,51,52,53,54,55,56,57,58,59]. Currently, the main kind of artificial intelligence techniques that could be used are machine learning (ML) and deep learning (DL). MLs are widely used in medical imaging and have proven to be brilliant tools to assist general clinical cancer research [27,28] and could be used considering radiomics metrics as input data. However, some of the ML algorithms are not capable of using unstructured data. DL is the best technique for analyzing unstructured data built by multiple representation learning models on raw data [29,30,31,32,33]. The radiomics hypothesis is that different imaging features could be used in diagnosis, in prognosis predicting and therapeutic response in different cancer types. In fact, radiomics features provide data on the tumor microenvironment that can relate to histologic grade, prognosis, response to treatment and survival [22,23,24,25,26]. The automation brought by radiomics analysis and artificial intelligence models offers the opportunity to enhance the radiologists qualitative judgment, therefore improving tasks such as tumor detection, volumetry delineation, segmentation of lesions, linking intralesional imaging characteristics to genotypes and prediction of outcomes.

The aim of this review was to investigate the application of radiomics analysis in cancer imaging with the particular aim to summarize the results of recent studies to support oncological imaging, specifically in regards to breast cancer, rectal cancer and primitive and secondary liver cancer. Furthermore, we have proposed suggestions to increase reproducibility and robustness in radiomics applications.

## 2. Materials and Methods

### 2.1. Literature Search

This review resulted in a self-contained study without protocol and without a registration number.

To select the studies for this informative review, different electronic databases were considered, such as: “PubMed (US National Library of Medicine, http://www.ncbi.nlm.nih.gov/pubmed, accessed on 16 December 2022), Scopus (Elsevier, http://www.scopus.com/, accessed on 16 December 2022) and Web of Science (Thomson Reuters, http://apps.webofknowledge.com/ accessed on 16 December 2022)”.

Articles published in the last four years were analyzed since this time window (January 2019–December 2022) and are consistent with the most recent developments and trends in the use of radiomics in oncology. For the paper search, the following keywords were combined: radiomics AND/OR rectal cancer/tumor AND/OR breast cancer AND/OR liver cancer/tumor/metastasis. Exclusion criteria was: (1) articles of radiomics in other fields different from cancer imaging; (2) type of article as commentary, updated article, editorial letter, review article meta-analysis or case report; (3) articles without sufficient information for consideration or if the paper did not provide the number of cases analyzed, the partitioning of the dataset, the segmentation method, the radiometric features extracted, the statistical model to evaluate performance or the analysis of findings in a quantitative form. Moreover, papers that were not written in the English language were excluded. PRISMA checklist [34] was used. The research was conducted according to PICOS model (population; interventions; comparator group; outcomes; study design—Table 1).

### 2.2. Data Extraction and Quality Analysis

**Figure 1:**
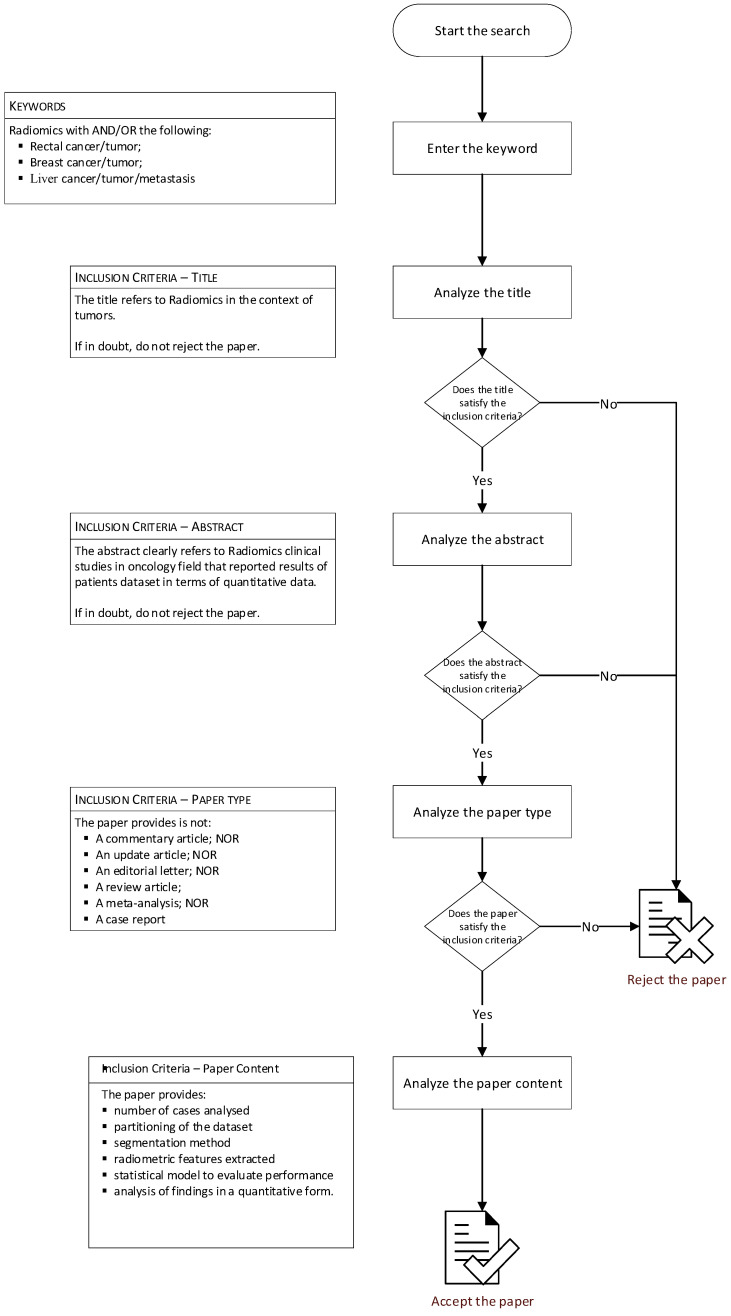
Flowchart of research methods.

Papers were selected by two investigators with over fifteen years of experience in radiomics analysis in cancer imaging (V.G. and R.F.) according to a specific procedure represented in the Figure 1. The two investigators performed data extraction and then recorded the outcome, field of application, number of cases analyzed, partitioning of the dataset, segmentation method, radiometric features extracted, feature selection approach and statistical model used to evaluate the performance of the extracted features and the paper results.

The methodological quality of each radiomics study was performed using the radiomic quality score (RQS) [35] by two different readers in consensus and by a third operator to solve disagreements between the two readers. The RQS includes 16 items that explore crucial steps of a radiomics pipeline: (1) image protocol quality; (2) multiple segmentations; (3) phantom study; (4) imaging at multiple time points; (5) feature reduction or adjustment for multiple testing; (6) multivariable analysis with non-radiomics features; (7) biologic correlates; (8) cut-off analyses; (9) discrimination statistics; (10) calibration statistics; (11) prospective studies registered in a trial database; (12) validation; (13) comparison to gold standard; (14) potential clinical applications; (15) cost-effectiveness analysis; (16) open science and data.

**Figure 2:**
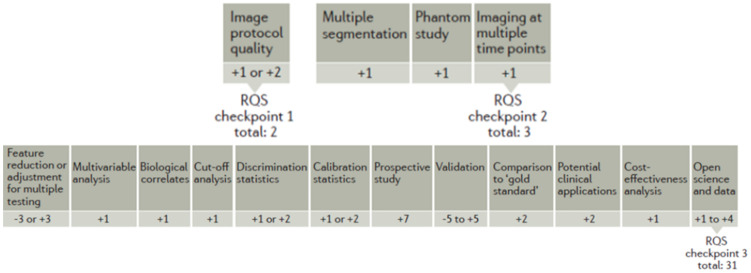
Radiomic quality score illustration.

Each of these items has a different weight and can contribute positively or negatively in terms of points attributed, with −8 being the minimum and 36 being the maximum score that can be reached. The absolute score is then converted to a percentage value (with 36 = 100%). Figure 2 illustrates the values that can be attributed to the 16 items to obtain the RQS.

## 3. Results

**Figure 3:**
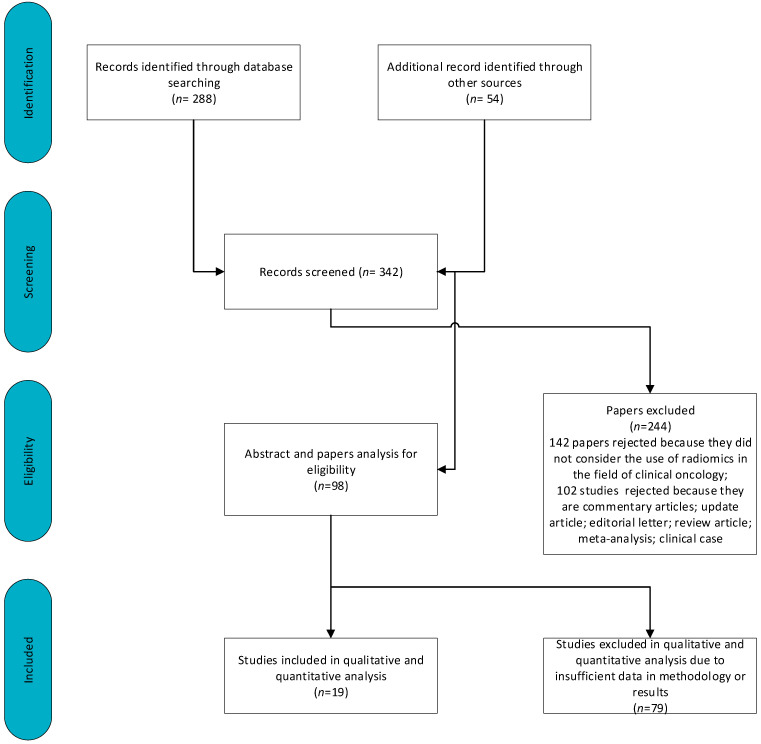
Schematic representation of included and excluded papers.

Figure 3 shows a schematic representation of the included and excluded manuscripts. There were 591 articles analyzed. Of these articles, 142 were rejected because they did not consider the use of radiomics in the field of clinical oncology. Another 158 studies were excluded because they were commentary articles, update articles, editorial letters, review articles, meta-analyses or clinical cases. A further 102 studies were excluded due to insufficient data in methodology or results.

Therefore, 19 manuscripts are included in this review. Table 2 reports the data collected by radiologists for these articles.

The studies included used features calculated by several imaging modalities including computed tomography (CT), positron emission tomography/CT (PET/CT), magnetic resonance imaging (MRI) and contrast-enhanced mammography (CEM) with different objective such as differential diagnosis, prognosis prediction, therapy assessment, etc. [9,11,13,22,23,36,37,38,39,40,46,47,48,49,50,53,54,58,59].

Table 1 reported the RQS assessment for each included study. The median RQS score was 15, which translates to 41.67% of the ideal score of 36. The lowest score was 8, which translates to 22.22% of the ideal quality score. Compared to the ideal score, the RQS of the following studies were the lowest in imaging at multiple time points: phantom study on all scanners, calibration statistics and cost-effectiveness analysis (0%), followed by multivariable analysis with non-radiomics features, prospective study registered in a trial database and validation. Seven studies with high score of seventeen (47.22% of the ideal quality score) [9,11,13,36,37,40,54]) earned additional points by using validation steps considering two datasets from two distinct institutes and open science and data domains (radiomics features calculated on a set of representative ROIs are open source).

### 3.1. Radiomics Studies in Rectal Cancer

Radiomics analysis in rectal cancer was used to assess and predict chemo-radiation therapy in locally advanced rectal cancer (LARC) patients using an MRI. It was also used in the prognosis prediction.

The five included studies in rectal cancer were retrospective studies (Table 3). The lowest score was 13, which is 36.11% of the ideal quality score. The highest score was 15, which is 41.67% of the ideal quality score. None of the included studies detected inter-scanner differences and vendor-dependent features, collected images of individuals at additional time points, reported calibration statistics, performed prospective studies registered in a trial database, performed a cost-effectiveness analysis report on the cost-effectiveness of the clinical application or made the code and data publicly available.

Xue et al. [46] demonstrated that the integrated model based on T2 weighted imaging and apparent diffusion coefficient maps had the potential for preoperative immunoscore expectations in rectal cancer. They found a model based on T2-weighted imaging and apparent diffusion coefficient images in the prognosis prediction and in the individualized immunotherapy, guiding the integrated model showed in the validation cohort an AUC of 0.768.

Chiloiro et al. [47] demonstrated that radiomics analysis achieved a good performance in identifying complete responders in rectal cancer and demonstrated that the diagnostic performance of radiomics improves when combined with standard clinical evaluation. Three models were produced: a radiomics model, a multidisciplinary tumor board model and a combined model that predicted with AUCs of 0.82, 0.73 and 0.84—the complete pathological response.

Cusumano et al. [48] investigated a MR radiomics model to detect complete pathologic response in LARC showing good performances both using 1.5 T and 3 T scanners. The predictive model AUC applied to the whole data set was 0.72, while values of 0.70 and 0.83 were obtained when the patient subgroups obtained with 1.5 T and 3 T MRI scanners were considered. Chiloiro et al. [49] supported a possible role of delta radiomics in predicting following occurrences of distant metastasis in patients with LARC. A logistic regression proved to be the best performing one with a testing set that balanced accuracy, sensitivity and specificity of 78.5%, 71.4% and 85.7%, respectively, to predict distant metastasis.

Chen et al. [50] reported that pre chemo radiation therapy MRI, post chemo radiation therapy MRI and delta radiomics-based models could predict tumor responses in LARC. The GLRLM-GLN calculated before therapy was able to classify pathological complete response groups with an accuracy at 88.5% on the training set and of 57.1% on the test set. When combined with 3D diameter, the accuracy increased on training data to 92.3%. The best predictors for a good response were the pre-global minimum combined with the clinical N stage in the multivariate analysis that obtained an accuracy of 100% on training and test sets.

The findings of these studies suggest that radiomics has the potential to provide valuable information in evaluating therapy and predicting prognosis in rectal cancer. However, there are still challenges in terms of standardization of imaging protocols, feature extraction and validation of radiomics models. Further research is needed to validate the clinical utility of radiomics and to establish its role in routine clinical practice.

### 3.2. Radiomics Studies in Breast Cancer

The usefulness of radiomics in distinguishing malignant from benign breast lesions as well as in predicting histopathological type, estimating tumor grade and assisting the staging procedure was explored in this manuscript. Therefore, the application of radiomics strategies as prediction tools for treatment response will be explored alongside the risk of recurrence.

The five included studies in breast cancer were conducted retrospectively (Table 4). The lowest score was 11, which is 30.56% of the ideal quality score, and the highest score was 17, which is 47.22% of the ideal quality score obtained published as open source extracted radiomics features [54]. None of the included studies detected inter-scanner differences and vendor-dependent features, collected images of individuals at additional time points, reported calibration statistics, performed prospective study registered in a trial database or performed an analysis report on the cost-effectiveness of the clinical application.

Fusco et al. [23] evaluated the possibility of using radiomics metrics by CEM and dynamic contrast enhanced MRI in the benign and malignant breast lesion discrimination through different classifiers performing balancing and feature selection procedures. The best performance was obtained considering 18 robust characteristics and a linear discriminant analysis with a precision of 0.84 and an AUC of 0.88.

Tsuchiya et al. [53] assessed the MRI-based radiomics model to differentiate phyllodes breast tumors from fibroadenomas, investigating several machine models. A support vector machine reached the best AUC of 0.96, and the combined model, which was constructed using both radiomics features and radiological features, had a significantly improved performance in the validation set (AUC of 0.97).

Petrillo et al. [54] used the CEM and the radiomics analysis in the classification of suspicious breast lesions and performed both univariate analysis and multivariate analysis to investigate the better approach and the higher accuracy in the classification of malignant and benign lesions. At univariate analysis, the best accuracy in the differentiation of benign and malignant breast lesions was obtained using the original_gldm_DependenceNonUniformity with an accuracy of 89%, while in the classification of the hormone receptor presence, a lower level of accuracy was found (81.65%). For multivariate analysis using features extracted from cranio-caudal images, the maximum test accuracy in the malignant and benign lesion differentiation was 96% with logistic regression. For features extracted from mediolateral oblique images, the best test accuracy was 92% and was always in the classification of breast lesions obtained using a classification tree algorithm.

Feng et al. [58] demonstrated that a radiomics feature set combining three DCE-MRI parametric maps and ADC maps yielded an area under the ROC curve of 0.839 within the training set and 0.795 within the independent validation set in breast cancer KI-67 determination.

Wang et al. [59] constructed a radiomics score significantly associated with disease-free survival (DFS) for locally advanced breast cancer (LABC) patients in training cohorts, validation cohorts and external validation cohorts (*p* < 0.001, *p* = 0.014 and *p* = 0.041, respectively). The radiomics-based nomogram showed better predictive performance of DFS compared with the TNM model. They demonstrated that radiomics scores could effectively predict the prognosis of LABC after neoadjuvant chemotherapy and radiotherapy.

### 3.3. Radiomics Studies in Liver Primitive and Secondary Cancer

The main potential applications of radiomic models in liver primitive and secondary carcinoma are to predict histology, predict response to treatment, predict genetic signature, predict recurrence and predict survival.

The nine included studies in liver primitive and secondary cancer were retrospective studies (Table 5). The lowest score was 8, which is 22% of the ideal quality score. The highest score was 17, which is 47.22% of the ideal quality score. None of the included studies detected inter-scanner differences and vendor-dependent features, collected images of individuals at additional time points, reported calibration statistics, performed prospective study registered in a trial database or performed an analysis report on the cost-effectiveness of the clinical application. However, the studies [9,11,13,36,37,40] earned additional points by using multivariable analysis with non-radiomics features or validation steps considering two datasets from two distinct institutes or open science and data domain published extracted radiomics features. 

Granata et al. [9] demonstrated that radiomics and machine learning analysis, based on the Gd-EOB-DTPA-enhanced magnetic resonance imaging (EOB-MRI) study, allow the identification of several biomarkers for detection of the different growth patterns in colorectal cancer liver metastases. Study [11] reported that using univariate analysis was not possible to accurately discriminate the RAS mutation status. Instead, considering a multivariate analysis and classification approaches, a k-nearest neighbors (KNN) exclusively with texture parameters as predictors achieved the best results (an accuracy of 87.5% with 91.7% of sensitivity and 83.3% of specificity on external validation cohort).

In another study, Granata et al. [13] confirmed that radiomics data can be used to detect several features that may have an impact on the treatment choice for patients with liver metastases, obtaining a more tailored approach. The radiomics metric Wavelet_HHL_glcm_Imc2 alone showed the best accuracy in discriminating between expansive and infiltrative tumor growth equal to 79%. Wavelet_LLL_firstorder_Mean showed the best accuracy in budding tumor detection equal to 86%, Original_firstorder_RobustMeanAbsoluteDeviation showed the best accuracy in identifying mucinous tumor types equal to 88% and Wavelet_HLH_glcm_Idmn showed the best accuracy in identifying tumor recurrence equal to 85%. The best linear regression model was achieved in the recurrence detection, combining linearly 16 radiomics metrics (accuracy of 97%). However, the best results were reached in the tumor front growth detection, combining seven radiomics features with an accuracy of 97%, a sensitivity of 90% and specificity of 100%. In addition, Granata et al. [36] investigated radiomics and ML approaches in the mucinous colorectal liver metastases evaluation by MRI, demonstrating that radiomics metrics could permit the characterization of the lesion subtype with a more tailored therapeutic approach. They showed that the best performance was obtained by T2-weighted combining linearly radiomics features using a linear regression (accuracy of 94%). Moreover, Granata et al. [37] demonstrated that the best performance in the discrimination of tumor budding was obtained by a KNN considering four radiomics predictors by T2-weighted MRI, yielding an accuracy of 93%, a sensitivity of 81% and a specificity of 97%. In all studies [9,13,36,37] the authors used multiple segmentations from different radiologists, an external validation dataset, detected and discussed biologic correlates and published as open data extracted radiomics features.

Yang et al. [22] established a predictive integrated model for early recurrence of hepato-cellular carcinoma (HCC) after ablation, and the model presented good predictive performance. Multivariate analyses suggested that the rad-score including four radiomics features, number of lesions, integrity of the capsule, pathological type and alpha-fetoprotein were independent influencing factors of HCC recurrence. The AUC of predicting early recurrence at 1, 2 and 3 years in the validation group was 0.72 (95% CI: 0.58–0.84), 0.61 (95% CI: 0.45–0.78) and 0.64 (95% CI: 0.40–0.87).

Gao et al. [38] investigated the ability of radiomics and deep features by MRI in the identification of a predictive model for the early recurrence of HCC after surgery. The authors integrated radiomics and deep features into a combined model and demonstrated improved performance in the detection of patients at high risk of early recurrence (area under curve (AUC) 0.840, accuracy 77.7%).

De Robertis et al. [39] demonstrated that CT texture analysis of pancreatic adenocarcinoma could identify features able to predict the liver metastases likelihood. This study included 220 patients. Tumor size, arterial HU_MAX, arterial GLZLM_SZHGE and portal GLCM_CORRELATION were significant predictors of the likelihood of liver metastases, with odds ratios of 1.1, 0.9, 1 and 1.49, respectively.

Shi et al. [40] reported that RAS and BRAF mutated tumors show discriminatory CT radiomic features which, if combined with semantic features, could help in the detection of tumors harboring RAS and BRAF mutations in patients with colorectal liver metastasis in order to increase patient stratification and customized treatments. The combined score of radiomic and semantic features could discriminate between wild-type and mutant patients with an AUC = 0.95 in primary cohorts and =0.79 in validation cohorts.

Radiomics studies have demonstrated its predictive value such as the grade of hepatocellular carcinoma, the ability to predict recurrence or the differential diagnosis of other primary or secondary liver tumors and the correlation with genetic mutations. However, once again the added value of radiomics in modifying therapeutic choices, and therefore as a decision-making model, is not clear.

## 4. Discussion

This manuscript summarizes recent radiomics studies in cancer imaging and discuss the challenges and limitations of the methodologies employed.

Radiomics looks to be very promising, as it has been applied in oncology to improve diagnosis and prognosis with the intention of helping the clinician, and is increasingly oriented towards precision medicine. The foundation on which radiomics is built is that imaging data indirectly carry significant information about tumor biology, behavior and pathophysiology, and can provide information that would otherwise not be apparent to purely visual radiological interpretation.

Radiomics presupposes an alternative non-invasive tool for characterizing tumors, which has experienced growing interest with the advent of more powerful and more sophisticated computer machine learning algorithms. However, the incorporation of radiomics into cancer clinical decision support systems still needs in-depth analysis of its relationship with tumor biology.

Moreover, there were many differences in the methods used in image segmentation, feature extraction and prediction model construction. Furthermore, some important aspects have not always been considered by the authors such as the importance of the external validation of the set in the evaluation of the intra and inter-observer variability and in the balancing of the data set. In fact, the critical problems in radiomics use are the insufficient standardization and generalization of radiomics results, data quality control, repeatability, reproducibility, database matching and model overfitting issues [55,57].

A key attention is to determine the availability of sufficient data to support the development of a radiomics signature. As a rule, for binary classification studies, one should aim to obtain at least 10–15 samples for each feature that is provided in the final radiomics signature [55,57].

In medicine, two different approaches can be applied to cancer imaging: radiomic features extracted from the target lesion that can be used as inputs for machine learning algorithms, or an entire medical image or a series of images to train a deep learning model to directly perform tumor detection, characterization and monitoring [60,61,62,63,64,65,66,67,68,69].

This review reported that radiomics metrics can extract biological and path-physiological evidence from target lesions from images, and the equivalent quantitative features can offer a precise non-invasive biomarker for oncological diagnosis, prognosis and outcome monitoring. Artificial intelligence has been used in conjunction with radiomics features to solve difficult problems that would have been intractable using traditional statistical approaches. In addition, artificial intelligence-based methods have made important advancements in the field of radiological oncological medical imaging [70,71].

However, great variability has been observed in methodologies for radiomics extraction, reduction and classification models. This aspect influences the reproducibility and generalizability of the results and a large level of variability can be observed in the scientific reports of the different authors. Many authors have not partitioned the data set. In general, the dataset should be divided into training datasets (70% of samples), test datasets (20% of samples) and validation datasets (10% of samples) [71]. Furthermore, the samples should also include external datasets to better validate the results of the radiomics and artificial intelligence procedures [72,73,74,75].

In addition, another major problem is the repeatability and replicability of artificial intelligence and radiomics techniques [76]. This problem is mainly linked to the variability of the image acquisition equipment and of the protocol itself, as well as to the variability of the reconstruction and pre-processing techniques of the images used, for example, to optimize the signal-to-noise ratio. Furthermore, image segmentation or feature extraction methods are not absolute and are problematic to apply to be completely standardized, which means that the implementation of several tasks simultaneously with deep learning and machine learning methods is still restricted [76,77,78,79,80,81,82,83,84,85,86,87,88,89,90,91,92,93,94,95,96,97,98,99,100,101].

To resolve this and to construct reproducible and standardized radiomics features extraction and building models, the following aspects should be considered: (1) to obtain reproducible radiomics features, image biomarker standardization initiative (IBSI) [77], a radiomics standardization initiative of the international community, should be considered and a common tool for feature extraction should be used to avoid lack of robustness and reproducibility in the steps of definition, implementation and pre-processing of images of radiomics features. (2) To build robust and generalizable models, the data can be enlarged by shearing, rotating or inverting original images.

Also, it is important to check if the data is balanced. When the proportions between classes are unequal in a classification problem, the data can be severely biased and a larger sample size may be required for the developed model to be generalizable. Data balancing techniques that include creating artificial data with algorithms such as the synthetic minority oversampling technique may be considered [40,78,79,80]. However, only a few papers have considered balancing techniques to reduce the unbalanced dataset problem [23].

In addition to the technical, regulatory and ethical requirements, problems need to be evaluated. As any big data project requires access to huge data sets, the collection of critical issues such as patient privacy and informed permission need to be addressed. This is critical not only by a medico-legal point of view, but also by a “human” point of view, because, like Coppola et al. [81] stressed, we must not superintend the meaning of the irreplaceable doctor–patient bond. Connecting doctors and patients directly will always be an important phase of healthcare services that artificial intelligence can never replace [2,20,68,102,103,104,105,106,107,108,109].

Among the studies analyzed, none assessed the differences between scanners and vendor-dependent characteristics, collected images of individuals at additional points in time, performed calibration statistics, represented a prospective study performed registered in a study database, conducted an analysis report on the cost-effectiveness of the clinical application or reported multivariable analysis with also non radiomics features. Seven studies reached a high score of 17 [9,11,13,36,37,40,54]) and earned additional points by using validation step considering two datasets from two distinct institutes and open science and data domains (radiomics features calculated on a set of representative ROIs are open source).

Our study has some limitations that should be taken into consideration. First, all studies included in this meta-analysis were retrospective in study design, which was subject to selection bias, underscoring the need for prospective validation. Second, in the absence of direct comparisons between radiomics models and other scoring or non-radiomics models, it is difficult to draw a conclusion that radiomics models are superior to other non-radiomics models. However, radiomics studies have had marked heterogeneity in their workflow. In the future, it will be necessary to establish and promote an imaging data acquisition protocol, standardize the research workflow, and conduct prospective multicenter quality control studies. Furthermore, combining radiomics with multiomics could lead to a breakthrough in the individualized medical treatment of tumors.

## 5. Conclusions

In conclusion, this research topic has involved many works which have made full use of radiomics in cancer imaging, but even in this case many aspects regarding the methodologies used should be considered. The potential of radiomics is becoming increasingly established, although there are still several aspects to be evaluated before the transition to routine clinical practice.

There are several challenges to address, including the need for standardization at all stages of workflow and the potential for cross-site validation using heterogeneous real-world datasets. Furthermore, multiple centers and prospective radiomics studies with more samples that add inter-scanner differences and vendor-dependent characteristics will be needed in the future, along with collected images of individuals at additional time points, reported calibration statistics and performed prospective studies registered in a study database.

## Figures and Tables

**Table 1:** Inclusion and exclusion criteria adopted to select studies according to the PICOS model.

PICOS	Inclusion Criteria	Exclusion Criteria
Population	Human studies	Non-human, animal or in vitro studies
Interventions	Radiomics in cancer imaging including rectal cancer, breast cancer and primitive and secondary liver cancer	Radiomics in another district of cancer imaging
Comparators	Not relevant	
Outcomes	Number of cases analyzed; partitioning of the dataset; segmentation method; radio-metric features extracted; statistical model to evaluate performance; analysis of findings in a quantitative form	Studies not reporting the defined outcomes
Study types	Retrospective or prospective	Guidelines, meta-analyses, systematic or narrative reviews, update articles, abstracts, letters, editorials, conference presentations and posters and case reports
Language	English	Non-English

**Table 2:** Radiomics quality score (RQS) assessment for all included articles.

Manuscript	Cancer District	Item 1	Item 2	Item 3	Item 4	Item 5	Item 6	Item 7	Item 8	Item 9	Item 10	Item 11	Item 12	Item 13	Item 14	Item 15	Item 16	RQS	%
Granata et al. [9]	Liver	1	1	0	0	3	0	1	1	2	0	0	4	1	2	0	1	17	47.22
Granata et al. [11]	Liver	1	1	0	0	3	0	1	1	2	0	0	4	1	2	0	0	17	47.22
Granata et al. [13]	Liver	1	1	0	0	3	0	1	1	2	0	0	4	1	2	0	0	17	47.22
Yang et al. [22]	Liver	1	0	0	0	3	1	0	1	2	0	0	2	1	2	0	0	13	36.11
Fusco et al. [23]	Breast	1	1	0	0	3	0	1	1	2	0	0	2	1	2	0	1	15	41.67
Granata et al. [36]	Liver	1	1	0	0	3	0	1	1	2	0	0	4	1	2	0	1	17	47.22
Granata et al. [37]	Liver	1	1	0	0	3	0	1	1	2	0	0	4	1	2	0	1	17	47.22
Gao et al. [38]	Liver	1	1	0	0	3	0	1	1	2	0	0	2	1	2	0	0	14	38.89
De Robertis et al. [39]	Liver	1	0	0	0	0	0	1	1	2	0	0	0	1	2	0	0	8	22.22
Shi et al. [40]	Liver	1	1	0	0	3	1	1	1	2	0	0	4	1	2	0	0	17	47.22
Xue et al. [46]	Rectal	1	1	0	0	3	0	1	1	2	0	0	2	1	2	0	0	14	38.89
Chiloiro et al. [47]	Rectal	1	1	0	0	3	1	1	1	2	0	0	0	1	2	0	0	13	36.11
Cusumano et al. [48]	Rectal	1	0	0	0	3	0	1	1	2	0	0	4	1	2	0	0	15	41.67
Chiloiro et al. [49]	Rectal	1	1	0	0	3	0	1	1	2	0	0	2	1	2	0	0	14	38.89
Chen et al. [50]	Rectal	1	0	0	0	3	1	1	1	2	0	0	2	1	2	0	0	14	38.89
Tsuchiya et al. [53]	Breast	1	0	0	0	3	0	1	1	2	0	0	0	1	2	0	0	11	30.56
Petrillo et al. [54]	Breast	1	1	0	0	3	0	1	1	2	0	0	4	1	2	0	1	17	47.22
Feng et al. [58]	Breast	1	1	0	0	3	1	1	1	2	0	0	2	1	2	0	0	15	41.67
Wang et al. [59]	Breast	1	1	0	0	3	0	0	1	2	0	0	2	1	2	0	0	13	36.11

**Table 3:** Radiomics Clinical Studies in rectal cancer.

Manuscript	Outcome and Application Field	Number of Analyzed Cases	Dataset Partition	Segmentation Method	Extracted Features	Feature Selection Approach	Statistical Model to Assess Performance
Xue et al. [46]	To establish and validate a radiomics model based on multi-sequence MR images for preoperative prediction of immunoscore in rectal cancer	A total of 133 patients	Randomly divided into training cohort (*n* = 92) and validation (*n* = 41) cohort according to a ratio of 7:3	The volumes of interest were manually delineated in the T2-weighted images and apparent diffusion coefficient images	A total of 804 radiomics features were extracted	Spearman correlation analysis and gradient boosting decision tree algorithm to select the strongest features	Multivariate logistic regression algorithm, including two single-mode models and two dual-mode models
Chiloiro et al. [47]	To investigate the contribution of radiomics analysis on post-treatment MRI for predicting complete pathological response after neoadjuvant chemoradiotherapy in locally advanced rectal cancer	A total of 144 LARC patients	any	A resident radiologist and radiation oncologist delineated the gross tumor volume on the axial oblique T2-weighted images	A total of 232 radiomics features were extracted belonging to statistical, morphological and textural families	Features selection was performed considering the predictive performance at the univariate analysis using the Wilcoxon–Mann–Whitney test and the Pearson correlation	A logistic regression model was developed to predict the treatment outcome
Cusumano et al. [48]	To develop a generalized radiomics model to predict pathologically complete responses after neoadjuvant chemoradiotherapy in patients with locally advanced rectal cancer	A total of 195 patients	The cohort from Internal Institution was 136 cases and the cohort from External Institution was 59 cases	Gross tumor volumes were delineated on the MR images	A total of 496 radiomic features were extracted after applying the intensity-based filter.	Features were standardized with Z-score normalization and an initial feature selection was conducted using Wilcoxon–Mann–Whitney test	Several logistic regression models combining the key features with a third one selected by those considered significant were elaborated and evaluated in terms of area under curve
Chiloiro et al. [49]	To study a correlation between the change in radiomic characteristics using pre- and post-neoadjuvant post-chemo-radio-therapy MRI with the rate of metastasis two years later (two years DM)	A total of 213 locally advanced rectal cancer patients were collected	The dataset was firstly randomly split into 90% training data and 10% testing data, for the validation	Gross tumor volumes were contoured by an abdominal radiologist and blindly reviewed by a radiation oncologist expert in rectal cancer	A total of 2606 features extracted from the pre- and post-chemo-radio-therapy gross tumor volumes were evaluated	Features selection was performed using a 5-folds cross-validation method	A total of 15 different classifiers were tested
Chen et al. [50]	To study radiomics features extracted from MRI scans performed before and after neoadjuvant chemoradiotherapy in predicting response of locally advanced rectal cancer	39 patients who underwent neoadjuvant chemo-radiation therapy for locally advanced rectal cancer were included	All patients were from a single center without external validation.	Segmentation was made segmented on the axial T2 weighted images with the open-source software tool IBEX by a radiation oncologist with specific expertise in rectal cancer	A total of 294 radiomic features were extracted, including shape, first-order, high-order texture and Laplacian of Gaussian filter-based features	After normalization, independent features were identified to reduce data dimension	Support vector machine based multivariate classification was used

**Table 4:** Radiomics Clinical Studies in breast cancer.

Manuscript	Outcome and Application Field	Number of Analyzed Cases	Dataset Partition	Segmentation Method	Extracted Features	Feature Selection Approach	Statistical Model to Assess Performance
Fusco et al. [23]	Differentiation between benign and malignant breast lesions using radiomic metrics from CEM and DCE-MRI images	A total of 44 patients with 79 histo-pathologically proven breast lesions	Dataset was divided in training and test set	Volume on interest segmented manually by two expert radiologists using Slicer3D	A total of 48 radiomics metrics using IBIS approach	A first selection of variables was made based on the results obtained from the univariate analysis: significant at nonparametric	Univariate and multivariate analyses were performed: non-parametric statistical test, receiver operating characteristic (ROC) and machine learning classifiers
Tsuchiya et al. [53]	To evaluate the diagnostic performance of MRI-based radiomics model for differentiating phyllodes tumors of the breast from fibroadenomas	A total of 88 patients	any	Manual segmentation	A total of 1070 texture features were extracted. Radiomic features were extracted from T2-weighted image, pre-contrast T1-weighted image and the first phase and late-phase dynamic contrast-enhanced MRIs.	A least absolute shrinkage and selection operator (LASSO) regression was performed to select features and build the radiomics model	A combined model was constructed using both radiomics features and radiological features. Machine learning classifications were conducted using support vector machine, extreme gradient boosting and random forest
Petrillo et al. [54]	To evaluate radiomics features to differentiate malignant versus benign lesions, predict low versus moderate and high grading, identify positive or negative hormone receptors and discriminate positive versus negative human epidermal growth factor receptor 2	A total of 182 patients	Dataset was divided in training and test set	Manual segmentation by two expert radiologists was performed using 3SSlicer	A total of 837 radiomics metrics were extracted by manually segmenting the region of interest from both craniocaudally (CC) and mediolateral oblique (MLO) views by Pyradiomics tool	Adaptive synthetic sampling balancing approach was used and a feature selection process was implemented.	Non-parametric Wilcoxon-Mann-Whitney test, receiver operating characteristic, logistic regression and tree-based machine learning algorithms were used
Feng et al. [58]	To evaluate a radiomics model dynamic contrast-enhanced magnetic resonance imaging parametric maps and apparent diffusion coefficient maps in the Ki-67 determination	A total of 205 patients	Patients were randomly divided into a training set (70% of patients) and a validation set (30% of patients)	Two radiologists with eight years and ten years of experience in breast MR imaging completed the layer-by-layer manual segmentation	A total of 946 radiomics features were extracted from each map by PyRadiomics	Significant radiomics features with *p* < 0.05 between patients with high versus low Ki-67 expression were first identified with the Mann–Whitney U-tests. Then, the least absolute shrinkage and selection operator was used	Support vector machine classifiers by combining different parameter maps and used 10-fold cross-validation to predict the expression level of Ki-67 were used
Wang et al. [59]	To predict survival outcome for locally advanced breast cancer patients and the association of radiomics with tumor heterogeneity and microenvironment	A total of 278 patients	Patients were randomly divided at a 1:1 ratio into training and validation cohorts	Region of interest of tumor was manually segmented along the lesion in every slice by the first reviewer and then reviewed by the second reviewer	Feature extraction was performed via 3D Slicer and its extension‚—slicer radiomics—derived from Pyradiomics	Features with both inter-observer and intraobserver ICC higher than 0.75 were selected for further analysis. LASSO regression is applied	Univariate and multivariate Cox proportional hazards model was applied

**Table 5:** Radiomics Clinical Studies in Liver primitive and secondary cancer.

Manuscript	Outcome and Application Field	Number of Analyzed Cases	Dataset Partition	Segmentation Method	Extracted Features	Feature Selection Approach	Statistical Model to Assess Performance
Granata et al. [9]	To assess radiomics and machine learning analysis in colorectal cancer liver metastases growth pattern	A total of 81 patients and 151 lesions	A training set of 51 patients with 121 liver metastases and an external validation set of 30 patients with a single lesion	The volume on interest segmented manually by two expert radiologists using Slicer3D	A total of 851 radiomics features were extracted using PyRadiomics package	A first selection of variables was made based on the results obtained from the univariate analysis: significant at nonparametric	Nonparametric test, univariate, linear regression analysis and patter recognition approaches were performed
Granata et al. [11]	To assess the association of RAS mutation status and radiomics-derived data by contrast enhanced-magnetic resonance imaging in liver metastases	A total of 76 patients with 130 liver metastases	The validation cohort consisted of a total of 24 patients among 76 patients.	Manual slice-by-slice segmentation was performed on each phase of VIBE T1-W images by two radiologists with fifteen years of experience on MR liver images	A total of 48 texture features by means of a package provided from MATLAB programming tools for radiomics analysis	The least absolute shrinkage and selection operator method was used to detect the robust features	Wilcoxon-Mann-Whitney U-test, receiver operating characteristic analysis, pattern recognition approaches with features selection approaches were considered
Granata et al. [13]	To evaluate the efficacy of radiological features by CT to predict histopathological outcomes after liver re-section in patients with colorectal liver metastases, assessing recurrence, mutational status, histopathological features (mucinous) and surgical resection mar gin	A total of 77 patients and 147 lesions	The internal training set included 49 patients and 119 liver colorectal metastases. The validation cohort consisted of 28 patients with single liver colorectal metastasis	The volume on interest segmented manually by two expert radiologists using Slicer3D	A total of 851 radiomics features were extracted using PyRadiomics package on CT portal phase.	A first selection of variables was made based on the results obtained from the univariate analysis: significant at nonparametric	Nonparametric Kruskal-Wallis tests, intraclass correlation, receiver operating characteristic analyses, linear regression modeling and pattern recognition methods (support vector machine, k-nearest neighbors, artificial neural network and decision tree) were considered
Yang et al. [22]	To investigate a model for predicting the early recurrence of hepatocellular carcinoma after ablation	A total of 181 patients with HCC	The training group was 119 cases; validation group was 62 cases	Radiologists manually delineated the region of interest along the edge of the lesion, layer by layer	LIFEx 4.90 software was used to extract radiomics features after delineating the VOI of each lesion, totally 200 for each patient.	The least absolute shrinkage and selection operator cox proportional hazards regression after univariate and multivariate analysis was used to screen radiomics features and build integrated models	Clinical information and image semantic features were added to construct combined model
Granata et al. [36]	To evaluate the radiomics and machine learning analysis based on MRI in the assessment of liver mucinous colorectal metastases	A total of 151 cases	The cohort of patients included a training set (121 cases) and an external validation set (30 cases)	The volume on interest segmented manually by two expert radiologists using Slicer3D	A total of 851 radiomics features were extracted as median values by means of the PyRadiomics tool on volume on interest according to IBSI initiative	A first selection of variables was made based on the results obtained from the univariate analysis: significant at nonparametric Kruskal–Wallis test and with an accuracy ≥80%.	Linear regression modelling and pattern recognition techniques including support vector machine, k-nearest neighbors, artificial neural network, and decision tree were performed to calculate the diagnostic performance considering the significant features
Granata et al. [37]	To assess the efficacy of radiomics features obtained by T2-weighted sequences to predict clinical outcomes following liver resection in colorectal liver metastases patients.	A total of 151 cases	The cohort of patients included a training set (121 cases) and an external validation set (30 cases)	The volume on interest segmented manually by two expert radiologists using Slicer3D	A total of 851 radiomics features were extracted as median values by means of the PyRadiomics tool on volume on interest according to IBSI initiative	A first selection of variables was made based on the results obtained from the univariate analysis: significant at nonparametric Kruskal–Wallis test and with an accuracy ≥80%	Linear regression modelling and pattern recognition techniques including support vector machine, k-nearest neighbors, artificial neural network and decision tree were performed to calculate the diagnostic performance considering the significant features
Gao et al. [38]	To develop a predictive model for postoperative early recurrence of HCC based on deep and radiomics features from multi-phasic magnetic resonance imaging	A total of 472 HCC patients	Training (*n* = 378) and validation (*n* = 94) cohorts	Three-dimensional segmentation of the whole tumor in all patients was manually performed on each phase usingITK-SNAP	A total of 864 radiomics features were extracted based on PyRadiomics.	The least absolute shrinkage and selection operator logistic regression algorithm for feature selection and model construction	Radiomics selected features and deep features were selected to construct a combined predictive model. With each model, through a linear combination of selected features, the predicted probability value of early hepatocellular carcinoma recurrence was calculated for a patient
De Robertis et al. [39]	To develop a predictive model for liver metastases in patients with pancreatic ductal adenocarcinoma	A total of 220 patients	Not reported	Tumor segmentation was performed manually using a software for medical image processing (LifeX)). Three regions of interest were drawn on the CT slice corresponding to the largest tumor diameter	A total of 39 textural features were automatically extracted from the ROIs	Non performed	Logistic regression model
Shi et al. [40]	To investigate whether radiomics and/or semantic features could improve the detection accuracy of RAS/BRAF gene mutation status in patients with colorectal liver metastasis	A total of 159 patients	A training set and a validation set were considered	Regions of interest in the portal venous phase CT images were segmented with a 3D semi-automatic segmentation method by two radiologists	A total of 2 semantic and 851 radiomics features were calculated	Features with an intraclass correlation coefficient or a concordance correlation coefficient lower than 0.75 were excluded for subsequent analysis	Seven machine learning methods were used to construct three scores predicting the gene mutation status, including artificial neural network, Gaussian, Bayes, k-nearest neighbors, support vector machine, logistic regression, AdaBoost, gradient boost classifier

## Data Availability

Data are available at link https://zenodo.org/records/10477650 (accessed on 13 November 2023).
